# A New Ecosystem-Based Cumulative Effects Assessment Framework to Enhance Strategic Environmental Assessment of Marine Spatial Plans

**DOI:** 10.1007/s00267-025-02231-0

**Published:** 2025-08-01

**Authors:** Virginia Morejón, Ainhoa González Del Campo, Ibon Galparsoro, Debbi Pedreschi

**Affiliations:** 1 School of Geography, University College Dublin, Co. Dublin, Ireland; 2 Marine Institute, Co. Galway, Ireland; 3 AZTI, Marine Research Division, Pasaia, Spain

**Keywords:** Marine planning, Marine environmental assessments, Impact assessments, Geographic information systems, Risk-based assessments, Ecosystem integrity

## Abstract

With the increase in marine spatial planning efforts the need for robust environmental assessments that account for multiple pressures of human activities on marine ecosystems is more critical than ever. However, Cumulative Effects Assessment (CEA) practice, a requirement of Strategic Environmental Assessment (SEA) of marine spatial plans, remains insufficient. This paper explores the integration of ecosystem-based approaches into SEA stages for holistic environmental assessments of marine spatial plans that prioritize ecological integrity. It also reviews advancements in marine CEA research, focusing on risk-based approaches for assessing cumulative effects, and addresses the existing disconnection between CEA science and environmental assessment practice. Emphasis is placed on improving key SEA stages that are critical to CEA by identifying principles and approaches that systematically and spatially address the interactions of various pressures and ecosystem receptors across the four dimensions (4D) of marine environments to assess cumulative effects risks. This novel approach, presents a holistic framework aimed at enhancing CEA practice within SEA of marine spatial plans, for more sustainable and ecosystem-focused planning outcomes in marine environments.

## Introduction

Marine spatial planning (MSP) efforts are gaining attention for their potential to support the Blue Economy by efficiently managing the allocation of ocean space among multi-sectoral activities, goods, and services, while also ensuring the health and productivity of marine ecosystems. Many European countries have developed national marine spatial plans outlining their vision for ensuring the sustainable use of their seas, aligning with the European Union (EU) Maritime Spatial Planning Directive (MSPD) 2014/89/EU (CEC 2014). The MSPD highlights the Strategic Environmental Assessment (SEA) Directive (2001/42/EC) as a key tool for evaluating the potential significant environmental effects of plan implementation and requires MSP to adopt an Ecosystem-Based Approach (EBA) (CEC 2014).

The rapid expansion of MSP can be considered an indicator of an expected increase in SEA and Environmental Impact Assessment (EIA) (CEC 2011) activity, along with Cumulative Effects Assessments^1^ (CEA)—a mandatory component of both. CEA is the process for systematically evaluating environmental changes resulting from the additive, synergistic, or antagonistic effects of multiple human actions and natural processes on ecosystem components—the species or habitats affected by different human activities and their pressures (Borja et al. 2016, 2024). Despite its critical role, CEA has long faced challenges in SEA practice (Weiland 2010; Gunn and Noble 2011; Duinker et al. 2013; Matome and Mulale 2023). Practitioners continue to struggle with inadequate knowledge and data for setting appropriate spatio-temporal boundaries (including consideration of transboundary effects), understanding the sensitivity^2^ (or vulnerability) of ecosystem components to interactive pressures with varying effects, and identifying clear thresholds or tipping points for ecosystem component responses (Therivel and Ross 2007; Pavlickova and Vyskupova 2015; Lally and González Del Campo 2019; Hague et al. 2022). These challenges, coupled with uncertainties surrounding climate change impacts, further complicate these assessments (Bhave et al. 2016; Gissi et al. 2021). The complexity is further exacerbated in offshore and deep-sea environments, where 4D (depth and time) considerations are even more relevant due to the movement of species across different depths over time, adding additional layers of difficulty to assessments (Galparsoro et al. 2024). These pose notable challenges for SEA and CEA within the context of MSP (Pinkau and Schiele 2021; Calado et al. 2022).

The legal requirements for marine conservation under various EU marine directives—such as designating marine protected areas under the Habitats and Birds Directives (CEC 1992, 2010), or achieving ‘good environmental status’ under the Marine Strategy Framework Directive (MSFD) (CEC 2008) and ‘good ecological status’ under the Water Framework Directive (WFD) (CEC 2000)—have driven increased research efforts on EBA for both MSP and CEA methods. EBA represents a shift to a more integrated and holistic approach to managing human activities, with the goal of balancing socio-economic and environmental objectives (Foley et al. 2010; Katsanevakis et al. 2011; Ansong et al. 2017; Haugen et al. 2024). This shift has led to the development of guiding principles to effectively implement EBA in MSP based on the Convention on Biological Diversity (CBD 2004).

While SEA has the potential to promote more holistic, ecosystem-based, and sustainable marine spatial plans in line with legal requirements, this potential remains underexplored and unrealized (Pinkau and Schiele 2021). Incorporating EBA principles can enhance SEA by providing the ‘ecosystem perspective’ often missing in practice, ensuring that environmental assessments comprehensively consider human-environment interlinkages within appropriate spatial and temporal boundaries (Gunn and Noble 2011; Declerck et al. 2022). Moreover, marine research can support SEA-CEA by addressing methodological limitations and providing data and insights to better understand and manage the complexities of marine environments. Risk-based CEA approaches have gained attention in marine research for evaluating the likelihood of interactions between human activities and/or pressures and ecosystem components, and their potential effects, rather than relying on deterministic impacts (Judd et al. 2015; Galparsoro et al. 2021; Brignon et al. 2022). Their ability to incorporate both quantitative and qualitative data has made them particularly useful when there is insufficient information on pressure-ecosystem relationships (Pedreschi et al. 2023; Piet et al. 2023).

**Fig. 1:**
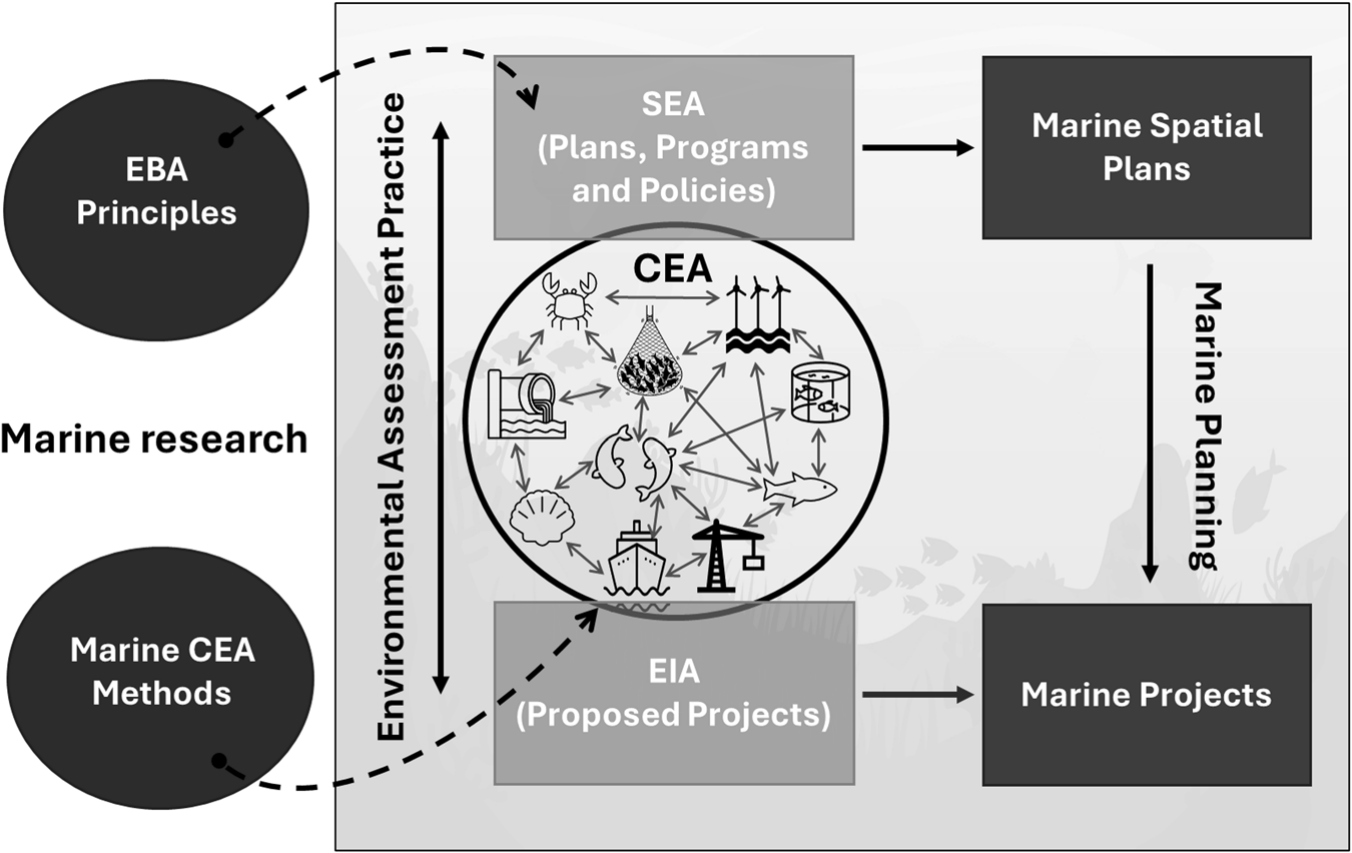
Illustration of the disconnect (indicated by the dotted arrows) between marine research (including ecosystem-based approaches (EBA) and Cumulative Effects Assessment (CEA) methods) and environmental assessment practice, highlighting the role of CEA in the evaluation of potential effects in Strategic Environmental Assessment (SEA) of Marine Spatial Plans, and within Environmental Impact Assessment (EIA) of Marine Projects (e.g., offshore energy development projects)

However, while a variety of risk-based methods have been developed in marine CEA research, there remains a disconnect (Fig. 1) between it and their practical application that needs to be addressed to strengthen SEA practice (Duinker et al. 2013; Noble 2015; Jones 2016; Blakley and Russell 2022). There is a need for more strategic and systematic methods to better evaluate cumulative effects in environmental assessments (Partidário 1996; Blakley and Russell 2022; Joseph et al. 2023). And in the context of the marine environment a regional approach for CEA in SEA is essential to account for the interconnectedness of coastal and marine environments. Furthermore, there is a need for structured frameworks that facilitate integration across the various approaches and methods with particular attention in spatially-explicit methods (e.g., use of Geographic information systems - GIS) that leverage the best available knowledge and data for capturing geographical variability in potential risks and impacts (González et al. 2011; Bragagnolo and Geneletti 2012; González and Geneletti 2021). In this context, the adoption of a ‘CEA mindset’ by both assessors and plan-makers, as noted by Sinclair et al. (2017), is essential for transitioning from conducting limited CEAs just to meet legal SEA requirements to using them to deepen our understanding of cumulative effects for more effective environmental assessments and better-informed plans.

In light of the existing potential to enhance SEA practice, this paper proposes a framework for an ecosystem-based SEA-CEA for MSP. It first examines how EBA principles can be integrated into the SEA process, highlighting the crucial role of CEA. It then explores advancements in marine CEA research to identify key methodological considerations for effective environmental assessments of marine spatial plans. The proposed framework aims to bridge the disconnect between CEA science and practice, promoting more holistic environmental assessments that can lead to sustainable MSP.

## Methods

### Integration of Ecosystem-based Approach Principles in SEA

While the MSPD mandates that plans adopt an EBA, it does not provide specific guidelines for implementation. To address this, the EBA operating principles proposed by Galparsoro et al. (2025) are used in this paper as the foundation for developing an ecosystem-based SEA framework (Table 1). These principles have been adapted from various guidelines on ecosystem-based approaches for specific application within MSP (e.g., Gilliland and Laffoley 2008; Piet et al. 2021b; Strosser et al. 2021a, 2021b). The potential contributions of EBA principles to key stages of SEA, specifically for enhancing CEA, are qualitatively examined. This analysis focuses on the scoping, baseline environment, Strategic Environmental Objectives (SEOs), and impact assessment stages of SEA, which prepare the groundwork for CEA (Bragagnolo and Geneletti 2012). The remaining SEA stages (i.e., alternatives, mitigation, monitoring) are outside the scope of this paper. While the ‘alternatives’ stage is subject to CEA and monitoring can help fill in data gaps and strengthen subsequent assessments, these stages largely rely on the outcomes of CEA rather than directly feeding into it. Principles focused on governance and management were not considered as they directly align with decision-making and plan implementation rather than environmental assessment and plan-making.

**Table 1:** Ecosystem-based approach to management principles adapted from Galparsoro et al. (2025)

Ecosystem-based Approach to Management Principles
**1. Use SMART (Specific, Measurable, Achievable, Realistic, and Time-bound) goals linked to targets and indicators to set balanced economic, social, and environmental objectives, which reflect societal choice**.
**2. Consider the ecological integrity, biodiversity, functioning, and resilience of marine ecosystems**.
3. Provide a framework to identify, conserve, and restore important components of coastal and marine ecosystems.
4. Account for the dynamic nature of ecosystems by setting long-term management objectives at appropriate scales.
5. Promote sustainability by maintaining ecosystem integrity to ensure the delivery of ecosystem services and socio-economic benefits.
**6. Include all marine uses and activities, considering their effects on resources and ecosystems at appropriate scales**.
**7. Understand ecosystem resistance, resilience, and cumulative human impacts to forecast ecosystem health under various climate-change scenarios**.
8. Use marine space efficiently to balance competing uses, minimize conflicts, and optimize co-use of compatible activities.
**9. Use of the best available information, including scientific and local knowledge, and use the precautionary principle when information is lacking**.
**10. Use a rigorous risk assessment and management framework for both natural and anthropogenic hazards**.
**11. Consider climate change uncertainties, ecosystem variability, external drivers, and interactions among human impacts on marine ecosystems**.
12. Establish a governance system to integrate policies and human activities affecting the marine ecosystems.
13. Promote efficient decision-making with economic, societal, and ecological incentives for marine protection and equity.
14. Recognize ecosystem connectivity and promote cooperation across regional and national boundaries.
15. Implement coherent planning with nested spatial scales and adaptive management.
16. Engage diverse stakeholders in all management stages with transparent communication.
17. Coordinate with other governance tools and policies for holistic management.

Principles highlighted in bold represent added value across different Strategic Environmental Assessment stages, including scoping, baseline environment, strategic environmental objectives, and impact assessment for enhancing Cumulative Effects Assessment

### Evaluation of Marine CEA Research

Existing published reviews were examined to identify the latest advancements in marine CEA, including Simeoni et al. (2023) covering marine CEA approaches from 2000 to March 2022 (30 papers) and Blakley and Russell (2022), covering CEA methods from 2008 to 2018 (11 papers related to marine CEA). More recent advancements were captured through a systematic search in Web of Science (WoS) and Scopus for the period from March 2022 to July 2024 using the query: (“cumulative effect assessment” OR “cumulative impact assessment”) AND (“marine” OR “coastal”). This search resulted in 28 documents from WoS and 26 from Scopus, from which 10 articles proposing new approaches were selected after a title, abstract, and methodology review. Additionally, 13 relevant papers were extracted from González et al. (2020) review of marine planning tools from 1998 to 2020. Reviews from OSPAR^3^ and the EU MSP Platform (2018) also helped identify another three reports from OSPAR and nine from the EU MSP. After removing duplicates, 75 papers were reviewed for their CEA methods. A first filter was applied to identify risk-based CEA approaches. Establishing cause-effect pathways (i.e., interactions) between activities, pressures, and ecosystem components is key for effective risk assessments and ultimately CEA (Judd et al. 2015; Stelzenmüller et al. 2018); therefore, papers not fitting this focus were excluded. The remaining 15 papers were reviewed for spatial specificity (central in the context of spatial planning) and support for EBA (e.g., by determining whether they address connectivity between multiple pressures-ecosystem components), narrowing it down to 10 that met all criteria. Finally, these methods were reviewed for their application in SEA and EIA practices, specifically for their ability to address current SEA-CEA challenges. The review examined their effectiveness in defining assessment boundaries and scope (including transboundary effects), evaluating interactions and diverse effect types (e.g., additive, synergistic, antagonistic, linear, non-linear), and addressing uncertainties related to climate change (see supplementary material).

## Results and Discussion

### Ecosystem-based Principles Enhancing Strategic Environmental Assessment

**Fig. 2:**
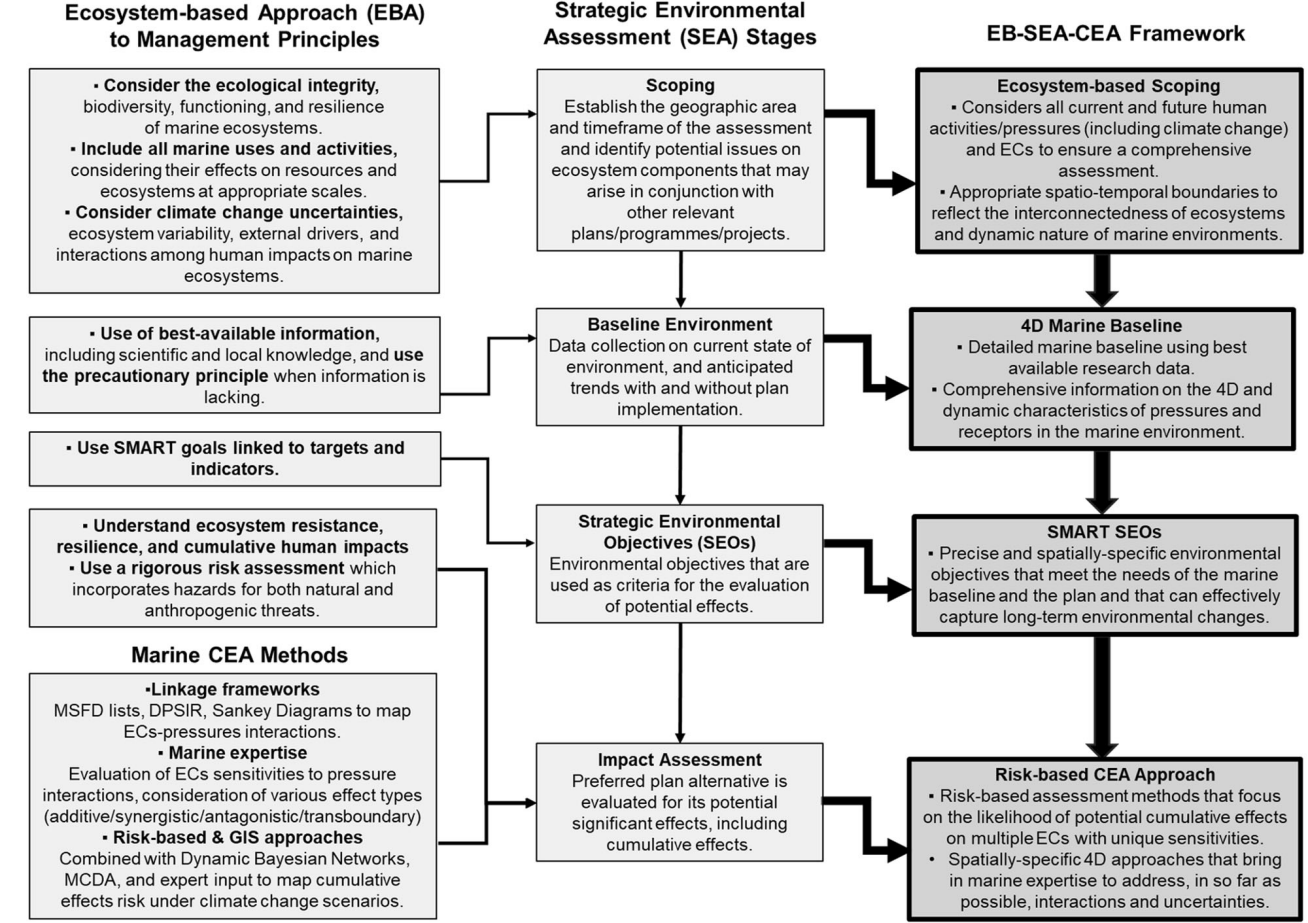
Integration of Ecosystem-based Approach (EBA) principles into Strategic Environmental Assessment (SEA) stages: scoping, baseline environment, Strategic Environmental Objectives (SEOs), and impact assessment, with methodological advancements from marine Cumulative Effects Assessment (CEA) methods to support a holistic, Ecosystem-based (EB)-SEA-CEA framework for Marine Spatial Planning. SMART Specific, Measurable, Achievable, Realistic, and Time-bound, MSFD Marine Strategy Framework Directive, DPSIR Drivers-Pressures-State-Impact-Response, MCDA Multicriteria Decision Criteria Analysis, ECs Ecosystem Components, 4D Four Dimensions

The qualitative exploration of EBA principles that can add value to CEA processes within SEA, identifies several considerations that can advance scoping, baseline, SEOs and impact assessment practice. Figure 2 presents the key findings of this exploratory literature review and defines the EBA principles that have the potential to strengthen these SEA stages (detailed below) and thus establish a stronger basis for a more robust and comprehensive CEA.

#### Scoping

The scoping stage is crucial in determining the assessment’s success (Bragagnolo and Geneletti 2012; Canter and Ross 2014; Morrison-Saunders et al. 2014; Polido and Ramos 2015). It defines the geographical, temporal, and assessment scope for evaluating the plan alternatives and identifies key issues by consulting with relevant environmental authorities. This stage involves understanding how the plan will interact with other existing and future plans and projects in the area, the implications of their potential effects, including cumulative and transboundary effects, and how climate change may exacerbate these conditions. However, setting appropriate spatial and temporal boundaries and relevant methodologies to capture all potential effects has proven to be challenging (Therivel and Ross 2007; Bragagnolo and Geneletti 2012; Bidstrup et al. 2016). Also, the absence of a ‘systems perspective’ often leads to oversimplified assessments that fail to consider how changes to one species can affect an entire ecosystem (e.g., through food web effects), resulting in inadequate assessment components that do not fully address the (potential) risk of ecosystem effects over time (Gunn and Noble 2011; Declerck et al. 2022). EBA principles, which highlight ecosystem interconnectedness, offer the potential to make the SEA scoping stage, including scoping consultations, more holistic. These include the consideration of the full ecological integrity, biodiversity, functioning, interconnectedness, and resilience of the marine ecosystem (Table 1, EBA principle 2). The prioritization of ecological integrity in SEA can enhance its role in balancing environmental objectives with development, recognizing and safeguarding well-functioning and highly connected ecosystems (both habitat and species) which are central to supporting multiple sectors and ecosystem services. Similarly, EBA principles that advocate for the inclusion of all current and future marine uses and activities (including climate change uncertainties), considering their potential direct, indirect and interactive effects on resources and ecosystem services at appropriate scales, and their interactions, are key (EBA principles 6 & 11). Comprehensive consideration of all marine uses and activities, the pressures they generate, ecosystem components, and the wider ecosystem upon which they depend, is needed to meaningfully inform the baseline stage. Setting up appropriate spatio-temporal scales during the scoping stage is crucial to ensure appropriate data are identified and/or collected for relevant maps to be produced. Finally, this SEA stage is also crucial for identifying knowledge and data gaps and addressing any uncertainties related to climate change conditions which can contribute to the spatio-temporal variability of pressures and ecosystem components.

#### Baseline environment

The baseline stage aims to provide a comprehensive picture of the current state of the environment and future trends (without plan implementation) focusing on the key issues identified during scoping. It requires examining ecosystem components across various topics such as biodiversity, human health, fauna, flora, water, air, and climate (CEC 2001). The resultant baseline forms the basis for selecting SEOs and assessing (cumulative) effects. Therefore, the baseline should offer detailed relevant information and data about high-sensitivity habitats and species (i.e., ecosystem components), including existing pressures from current plans, projects, or activities, and expected interactions with pressures resulting from the proposed plan (DHLGH 2022). Although the use of best-available knowledge and the precautionary principle are mandated by the SEA Directive, EBA principles can help reinforce this (Table 1, EBA principle 9). This principle emphasizes the need to transparently capture what is known/unknown, what needs to be known, and how to proceed in the face of uncertainty.

The baseline stage should be strongly informed by the scoping stage EBA principles on ecological integrity, comprehensive consideration of marine uses and activities, and climate change. This is crucial to ensure that SEAs move beyond the static representation of baseline conditions, commonly used to describe biodiversity in environmental assessment practice and conservation management (Partidário 2012; Gillon et al. 2016; Kovac et al. 2018), and move towards baselines that capture the fluid and dynamic nature of coastal and marine environments. This includes capturing the full range of ecosystem components, from static benthic habitats to highly mobile marine wildlife, and recognizing that baseline/current conditions encompass a wide range of pressures and impacts arising from both marine and land-based human activities. It also requires the consideration of potential interactions with additional future pressures within the portfolio of existing pressures affecting the ecosystem.

The MSFD lists key activities, pressures, and impacts on ecosystems that should be considered for management (CEC 2008), and valuable datasets have been produced to support these efforts (e.g., EEA 2020, OSPAR 2023a, b). Additionally, the EU Marine Observation and Data Network (EMODnet) portal (www.emodnet.eu) centralizes marine data from numerous organizations, focusing on human activities. These data improve marine baseline information and provide clearer insights into the spatio-temporal characteristics of pressures, which are often underrepresented in SEAs (González 2008; González et al. 2011). Also, the OSPAR Assessment Portal (https://www.ospar.org) provides thematic assessments that can provide valuable information on indicators and thresholds for different ecosystem components and pressures. Enhanced data sharing and utilization, including greater involvement of marine scientific expertise and local knowledge in SEA processes, are also crucial for building more comprehensive and accurate marine baselines in SEA practice. This also helps identify gaps in data and inform subsequent stages about the associated uncertainties and guiding future decisions towards a precautionary principle.

#### Strategic environmental objectives (SEOs)

The wider scope of SEA often results in less detailed baselines, or a lack of specificity with regards to thresholds/tipping points needed for effective and robust assessments (Therivel and Ross 2007). To address this, some SEAs use SEOs to structure assessments, providing a framework for evaluating how a plan’s objectives align with or deviate from these objectives. While SEOs are not a formal requirement under the SEA Directive, they align with the Directive’s requirement to formulate environmental objectives and are widely adopted in Ireland and the UK (González 2008; Ravn Boess 2024). SEOs are developed for each environmental topic (e.g., biodiversity or human health) and are often derived from objectives, targets, and indicators established in local policies, and/or global strategies, such as Sustainable Development Goals (UN 2015). Nevertheless, SEOs often lack the specificity needed to effectively measure how a plan contributes to or detracts from environmental objectives over the long term (González 2008). The EBA principle (Table 1, EBA principle 1) that advocates for SMART—Specific, Measurable, Achievable, Realistic, and Time-bound—objectives linked to targets and indicators, can help address this by facilitating the denotation, spatial-specificity and trackability of SEOs.

Since SEAs set the rules for development consent (Therivel and Ross 2007), they must offer specific and realistic guidance on how future development should proceed to achieve these goals. Adopting SMART objectives can ensure that SEOs are more precise, targeted and efficient, particularly when they are closely aligned with the current state of the baseline. In this context, as noted by González (2008), spatial data from baseline maps can play a crucial role in shaping SEOs by incorporating spatial dimensions, and GIS can further assist by visualizing and prioritizing areas that need specific attention. For example, if an SEO focuses on achieving environmental objectives under the MSFD or WFD, the SEA should establish which particular objectives apply in which location/context. This ensures the marine baseline accurately reflects the various geographical areas within the assessment’s boundaries, including relevant pressures and ecosystem components thresholds or tipping points within the plan’s timescale. This approach would enable SEOs to be tailored not only to broader environmental goals, but also to the specific scope of the plan/program/policy, thus enhancing their relevance and making them more operational.

#### Impact assessment

The impact assessment stage is arguably the most critical part of a SEA process, as it evaluates the potential effects of plans/programs/policies alternatives, followed by a more detailed examination of the preferred alternative (informed by scoping, baseline, and SEOs) to understand and guide the long-term use and management of the implementation area. Traditionally, SEA impact assessments have relied on SEO-based matrix-based methods (González and Geneletti 2021; DHLGH 2022). While matrices provide a clear overview at a strategic level, they fall short in capturing the complex spatio-temporal distribution of effects, essential for a robust CEA in SEA (González 2008; González 2012). Current SEAs have been limited in their ability to identify specific locations where effects might occur or their varying impacts on interconnected ecosystem components with particular sensitivities. These limitations highlight the need for improved environmental assessment methods that offer a more comprehensive, spatially-explicit, and quantifiable understanding of potential direct and indirect cumulative effects (González 2008; González et al. 2011).

The adoption of EBA principles, specifically principles 7 and 10 from Table 1, can help in addressing some of these methodological shortcomings. These principles focus on understanding ecosystem resistance, resilience, along with cumulative human impacts and environmental drivers, to predict ecosystem health. They also advocate for the use of risk assessment and management frameworks to identify and quantify social, economic, and ecological risks and trade-offs. Incorporating these principles can highlight the need for CEA methods that enhance the understanding of the specific sensitivity of different ecosystem components to pressures, including their unique behaviors and variations across spatial and temporal scales. The joint consideration of pressures, ecosystem components, and their interrelations is often insufficiently addressed in SEAs for MSP (Pinkau and Schiele 2021; Declerck et al. 2022), yet is essential for holistic assessments (Dubé et al. 2013). Risk-based approaches have been considered valuable for addressing these complexities and uncertainties and are also recommended by the MSFD (Brignon et al. 2022; Declerck et al. 2023). Therefore, it is crucial to explore how such methods could improve CEA within SEA practice. The following section further examines the specific characteristics of advanced CEA approaches towards better-informed planning decisions.

### Marine CEA Methods: Opportunities to Improve SEA Practice

Many CEA methods developed in marine research are based on the cumulative impact mapping approach developed by Halpern et al. (2008). This is a straightforward yet effective spatial overlap analysis for quantifying cumulative impacts by (1) mapping the spatial distribution and intensity (scale, frequency, functional impact) of each anthropogenic activity (e.g., fishing efforts); (2) mapping the spatial distribution of marine receptors (e.g., coral reefs); (3) deriving weighting factors to reflect the vulnerability (resistance and recovery time) of each ecosystem receptor to the intensity of activities and (4) aggregating across to produce a cumulative impact score for a single grid cell. This approach has been further developed to enable cumulative impacts scenario mapping (e.g., Symphony; Hammar et al. 2020), and more recently, integrate risk assessment (e.g., SCAIRM – Piet et al. 2023). All of these methods are supported by extensive expert input, but methodological challenges remain (such as scale and pressure/ecosystem interaction knowledge gaps and data limitations). In the context of this paper, and the importance of a risk-based approach to CEA, more advanced methods that account for risk considerations were reviewed and are discussed next.

#### Assessment scope and boundaries

Most of the reviewed papers define their assessment scope using Halpern’s et al. (2008) mapping approach of activities/pressures and ecosystem components by identifying key components within a case study area through expert consultation. However, a key limitation of this method is its static nature, which overlooks dynamic aspects of cumulative effects in the marine environment, such as pressure dispersal across the water column and transboundary impacts—critical factors when setting boundaries for impact assessments. To address this, a limited number of studies have incorporated additional factors that account for the 4D dimensions of coastal and marine environments. For example, some studies use hydrodynamic modeling for pressures (e.g., Menegon et al. 2018a, 2018b), and others evaluate pressure dispersal as part of risk assessments (e.g., Piet et al. 2023). Such methods are particularly valuable in cases where there is no obvious spatial overlap between human activities and ecosystem components, but the pressures may still affect the ecosystem components. Time, however, remains a less explored aspect of cumulative effects (Willsteed et al. 2017). For instance, Battista et al. (2017) apply a temporally static model, potentially limiting its use in the context of evolving environmental changes, such as intensified climate change. While spatio-temporal variability of ecosystem components and pressures is often discussed and assessed, for instance through temporal overlap (i.e., when ecosystem components and pressures are likely to co-occur), the need for more robust models that accurately reflect ecosystem and climate change dynamics is increasingly recognized (Declerck et al. 2022, 2023).

#### Addressing interactions

Understanding human and environment interactions begins with a thorough assessment of activities/pressures and receptors/ecosystem components, commonly in consultation with marine experts (e.g., Battista et al. 2017; Piet et al. 2023). From a CEA risk assessment perspective, establishing linkages between these components is crucial (Judd et al. 2015; Muñoz et al. 2018; Stelzenmüller et al. 2018). Some of the reviewed methods support expert consultation with predefined lists of activities and ecosystem components from the MSFD and use visual tools to establish linkages or impact chains. For instance, they use network analyses such as the Drivers-Pressures-State-Impact-Response (DPSIR) framework (e.g., Robinson et al. 2014; Furlan et al. 2020), or Sankey Diagrams (e.g., Menegon et al. 2018a) to build linkages and graphically represent pathways and potential interactions. Given current knowledge limitations and the scarcity of pressure-impact and receptor-vulnerability spatial data available, interactions are challenging to identify. The majority of reviewed methods address only additive effects, as synergistic effects across multiple pressures remain difficult to evaluate, and antagonistic effects are often under-considered. However, some approaches, such as those by Battista et al. (2017), have begun to tackle these complexities by incorporating expert judgment to quantify synergistic and antagonistic effects through the use of an “additional threat modification factor” in pressure-receptor evaluations. Furlan et al. (2019) offer insights into spatial modeling of pressures by deriving GIS-based maps of spatial pressure intensity distribution, building on Furlan et al. (2018). They use a combination of ‘Choquet integral’^4^ techniques and advanced multicriteria decision analysis (MCDA) to spatially evaluate pressure interactions and estimate potential antagonistic and synergistic effects from multi-hazard interactions. Other methods use the number of links per pressure-receptor to indicate the likelihood of synergistic or antagonistic effects, but given the limited understanding of these interactions, the authors recommend interpreting outputs with caution (e.g., Piet et al. 2023).

#### Accounting for impact types and distinct effects

Cumulative effects can result from a combination of direct and indirect impacts, with both linear and non-linear consequences on ecosystem components and ecosystems. Risk-based approaches assess the likelihood of these effects by evaluating pressure intensity and the specific sensitivity of an ecosystem component to such pressures (Stelzenmüller et al. 2010). Most reviewed methods (largely based on the Halpern et al. (2008) approach) consider factors such as resistance, recovery time, scale, frequency, and intensity of pressures in their assessments. More recent risk-based methods build on this foundation, using similar principles (though terminology may vary across methods, such as recovery time vs. resilience) while refining techniques to deepen insights into pressure-ecosystem component interactions and inform management. For instance, impact chains created through tools like DPSIR or Sankey Diagrams are evaluated with marine expertise, using categorical risk-based scoring systems from other studies to weigh the contribution of each link and prioritize management objectives on the greatest threats (e.g., Robinson et al. 2014). Other methods also incorporate additional parameters such as magnitude, hazard, and receptor behavior (e.g., ability of receptors to evade pressures), to provide more realistic sensitivity scores that reflect the unique vulnerabilities of ecosystem components to pressures (e.g., Furlan et al. 2019, 2020; Piet et al. 2023). Battista et al. (2017) further includes attributes like habitat connectivity and food web structure in sensitivity assessments to address direct and indirect impacts, as well as cascading effects. However, the focus of the majority of the reviewed papers remains on direct, linear, and negative impacts. Overall, assessing more complex indirect and non-linear impacts, such as regime changes (due to exceeding tolerance levels, thresholds, and/or tipping points), or food-web changes with cascading effects across various pressure-ecosystem interactions, remains a significant challenge.

#### Integration of climate change and uncertainties

Climate change is frequently treated as an additional pressure, using spatial layers such as sea surface temperature, ocean acidification, or UV radiation (Halpern et al. 2008). More recent approaches have investigated how climate change may interact with and/or amplify other pressures, including modeling multiple climate change scenarios over longer timeframes (Battista et al. 2017; Furlan et al. 2019). However, future scenarios of climate change and human development are inherently uncertain and hard to predict. Limitations in scientific data and knowledge underpinning cumulative effects interaction pathways and magnitude increase this uncertainty. Probabilistic models like Bayesian Networks are potentially valuable tools for adaptive marine management, as they can effectively incorporate related uncertainties (e.g., Furlan et al. 2020). Dynamic Bayesian Networks in particular can be especially useful for addressing uncertainties across different planning scenarios as they can incorporate temporal dimensions in the analysis (e.g., Furlan et al. 2020; Declerck et al. 2022).

#### Spatial approaches

Stelzenmüller et al. (2018) noted that GIS has been widely used to support spatially-explicit CEA. It remains essential for visualizing impacts (Depellegrin 2016), and combining it with tools like MCDA, Bayesian Networks, and expert input can significantly enhance CEA outputs (e.g., Furlan et al. 2019, 2020; Declerck et al. 2022). For instance, Furlan et al. (2020) demonstrated the effectiveness of integrating GIS’s visualization capabilities with the modeling and prediction functions of Bayesian Networks. Such GIS-based Bayesian Network framework supports evaluating the probability and uncertainty of cumulative effects under various ‘what-if’ scenarios, including different climate change conditions. Moreover, Furlan et al. (2019) and Declerck et al. (2022) have emphasized that combining GIS with Dynamic Bayesian Networks can be a powerful approach for evaluating cascading effects and spatio-temporal dynamics in marine environments. This combination can allow for the analysis of how relationships and impacts evolve over time, which is crucial for managing risks that vary across space and time. Declerck et al. (2022, 2023) also advocate for increased monitoring efforts to generate the spatial data required to enable their routine use for CEA.

#### SEA or EIA application

The reviewed CEA methods have been predominantly developed for marine management and MSP, with limited focus on SEA and EIA, suggesting a disconnect between marine research and environmental assessment practice. While some methods claim to support SEA and EIA by centralizing relevant marine data (e.g., Menegon et al. 2018b), they primarily focus on specific MSP stages and do not elaborate on their application in SEA or EIA stages. Of the reviewed papers, the only ones specifically focused on enhancing CEA within SEA were Declerck et al. (2022, 2023). They stress the need for better alignment between CEA efforts in SEA and MSFD, advocating for an approach that integrates the ecosystem-based approach defined by OSPAR and the MSFD into SEA and EIA for a more holistic assessment framework at the European level.

### Proposed Ecosystem-based SEA-CEA Framework

Leveraging the key scientific advancements in marine CEA approaches, and adopting EBA principles, has clear potential to enhance and improve holistic SEA. This section introduces a framework that supports and encourages a ‘CEA mindset’ from the outset, promoting ecosystem-based decision-making (Fig. 2).

The following components outline the structure of the proposed framework, and a series of associated recommendations, for advancing SEA-CEA practice for MSP:

- **Ecosystem-based scoping**: For an effective CEA, this stage must recognize the interconnected nature of ecosystem components. Although ecosystem components vary across space, time, and depth in the marine environment, they remain integral parts of a cohesive ecosystem. As such, considering the ecological integrity of ecosystem components when assessing potential significant impacts arising from the proposed plan is important. For example, if the plan involves activities that disturb benthic habitats, it is essential to consider not only the species directly affected but also those that rely on these habitats for food or shelter. The scoping process must account for all relevant activities and pressures (both existing and introduced by the plan) to comprehensively address potential issues on a range of ecosystem components (from static habitats to highly mobile species) and their connectivity (the ecosystems that they rely on) over space and time, including climate change. This includes considering pressures that may disperse beyond boundaries and across the water column (e.g., contaminants) or those occurring at different times of the year (e.g., changes in sea surface temperature). Furthermore, scoping for climate change must identify specific factors (e.g., sea surface temperature, ocean acidification) and their spatio-temporal dynamics, as well as how they may interact with existing and future pressures, potentially worsening conditions for ecosystem components. By taking these considerations into account, effective assessment boundaries can be set to reflect the variability of all factors across different temporal (e.g., seasonal, annual, long-term) and spatial (e.g., local, regional, global) scales within the complex 4D marine environment. Given the intricacy of this process, it is crucial to involve marine experts and incorporate both scientific and local knowledge at the SEA scoping stage to ensure no significant pressures or ecosystem components variations are overlooked.
- **4D marine baseline**: This stage is about using the best available marine data within an ecosystem-based scoping process to create detailed and dynamic marine baseline maps. These maps should capture the 4D nature of the marine environment. The aim should be to depict variations within the water column, illustrating different habitats/species across various depths, to enable the identification of overlaps in three-dimensional space and temporal sequences with specific pressures. It is essential that such maps include not only the spatial distribution of (existing/proposed) activities and key ecological components but also their associated pressures and sensitivities, respectively, which are often underrepresented in SEA baseline maps. Therefore, the marine baseline maps should provide a clear snapshot or ‘inventory’ of the state of ecosystems before plan implementation to enable effective monitoring of changes over time. This monitoring is critical for identifying impacts on both mobile and immobile ecosystem components, which may be directly or indirectly affected by various pressures—whether these arise from plan’s activities, environmental drivers, or climate change. Using the most relevant data sources and confirming that these are fit for the scale and purpose of the assessment is key. Additionally, incorporating data on indicators, thresholds and tipping points (e.g. from the OSPAR thematic assessments) into the maps wherever possible is critical for informing the development of targeted SEOs. Finally, the marine baseline should identify knowledge and data gaps, ensuring that uncertainties are addressed where possible, and made explicit where they persist, to ensure the application of the precautionary principle in decision-making.
- **SMART SEOs**: For SEOs to effectively support CEA, they need to be SMART and spatially explicit. SEOs should be informed by the ecosystem-based scoping and marine baseline stages to accurately reflect the varying environmental and socio-economic conditions within the plan area and the assessment needs. To achieve this, SEOs should include spatially-explicit and realistic indicators or thresholds/tipping points that should not be exceeded to ensure the protection of habitats or species at risk of being affected by plan implementation. For example, if an objective aims to meet MSFD targets related to non-indigenous species, the available spatial data should be used to map their current state and understand their relevance to the area where the plan will be applied. Ensuring that SEOs are specific and contextually relevant can help address both local and global ecosystem conservation goals more effectively.
- **A Risk-based CEA approach**: This framework proposes adopting a holistic risk-based approach to assess the likelihood of cumulative effects from various pressures (including climate change) on interconnected ecosystem components with unique sensitivities. Given the significant uncertainty and limited empirical data available (especially regarding pressure-ecosystem component interactions), a risk-based approach is particularly relevant for SEAs in marine spatial plans, as they must anticipate the adverse effects of future plans and subsequent project implementation, which may involve unknown pressures and/or sensitivities that are not fully understood. Adopting risk-based methods is essential for establishing cause-effect pathways, assessing the likelihood of interactions, and understanding the unique sensitivities of various ecosystem components to pressures of differing intensities. While it is acknowledged that there are no one-fits-all methods, using supporting tools when consulting marine expertise, such as DPSIR or Sankey diagrams based on the MSFD lists of activities, pressures, and ecosystem components, is vital for ensuring that SEA aligns with MSFD objectives. Such linkage tools can be useful for a relatively quick visualization of the top sectors contributing pressures to the region and any potential for synergistic or antagonistic effects that may result from the plan. The resulting linkage diagrams can form the basis for the subsequent risk assessment analyses and/or spatial mapping of overlaps to evaluate if a marine spatial plan will affect MSFD objectives across different geographic areas. Spatial overlap analysis (e.g., Halpern et al. 2008) can be a valuable approach for visualizing the spatio-temporal variability of pressures and ecosystem components and can help identify potential transboundary effects (e.g., by integrating pressure dispersal assessments (Piet et al. 2023) or applying spatial pressure modeling techniques (Furlan et al. 2019)). Given that GIS remains the preferred tool for visualizing impacts, as a spatially-explicit method which supports location decisions in MSP, it is essential to explore its use in combination with advanced modeling methods like Dynamic Bayesian Networks, and MCDA. These methods are effective for evaluating interactions across spatial and temporal scales under various climate change scenarios. The specific linkage framework, risk assessment and/or spatially-explicit analytical methods selected should reflect the available data and knowledge needs, and their ability to manage uncertainties, particularly in regions with limited data.

## Conclusion

The recognition of the need to progress toward ecosystem-based management and planning approaches calls for a more holistic, ecosystems-level perspective in SEA, where the role of CEA is crucial. There is an urgent need to adopt CEA methods in SEA that are specifically designed to evaluate the potential effects of marine spatial plans on ecosystems, particularly those with unique sensitivities in complex and rapidly changing ocean environments—an area where current SEA practices often fall short. Clear links between EBA principles and the SEA process have been identified, and state-of-the-art methods for assessing cumulative effects in the marine environment have been defined. These developments have led to a new framework that enhances marine SEA practice by integrating EBA principles and CEA approaches driven by marine research, bridging the disconnect between CEA science and environmental assessment practice. The framework proposed in this paper aims to strengthen each of the SEA stages that feed into CEA, gradually building a holistic systems-perspective from scoping to impact assessment, ensuring that SEA remains aligned with current state-of-the-art methods for environmental assessments in the marine environment, and leading to better evaluation of potential effects of MSP efforts. This will contribute to ensuring a better evolving understanding of cumulative effects and, by extension, to more robust CEA processes and outputs that effectively inform other relevant SEA stages such as mitigation and monitoring. The framework is also applicable to onshore planning contexts and has the potential to enhance overall SEA practice.

## Supplementary information

CEA methods evaluation criteria

## Data Availability

No datasets were generated or analyzed during the current study.

## Footnotes

1 Cumulative Effects Assessment (CEA) and Cumulative Impact Assessment (CIA), are used interchangeably in the literature, but Piet et al. (2021a) differentiate them by defining impact as the result of pressure-induced effects. For consistency, this paper will use CEA.

2 Sensitivity refers to the degree to which a system can be affected based on its intrinsic characteristics. Vulnerability is the likelihood that a system will be exposed to a pressure and be sensitive to it (Zacharias anf Gregr 2005).

3 The OSPAR Convention is the current legislative instrument that regulates international cooperation on marine environmental protection in the North-East Atlantic.

4 The Choquet integral is a discrete fuzzy integral that generalizes additive operators like the ordered weighted mean. It is well suited for situations with synergistic or antagonistic effects among the criteria being aggregated (Zabeo et al. 2010).
